# Long-range temporal correlations in scale-free neuromorphic networks

**DOI:** 10.1162/netn_a_00128

**Published:** 2020-04-01

**Authors:** Shota Shirai, Susant Kumar Acharya, Saurabh Kumar Bose, Joshua Brian Mallinson, Edoardo Galli, Matthew D. Pike, Matthew D. Arnold, Simon Anthony Brown

**Affiliations:** The MacDiarmid Institute for Advanced Materials and Nanotechnology, School of Physical and Chemical Sciences, Te Kura Matū, University of Canterbury, Christchurch, New Zealand; The MacDiarmid Institute for Advanced Materials and Nanotechnology, School of Physical and Chemical Sciences, Te Kura Matū, University of Canterbury, Christchurch, New Zealand; The MacDiarmid Institute for Advanced Materials and Nanotechnology, School of Physical and Chemical Sciences, Te Kura Matū, University of Canterbury, Christchurch, New Zealand; The MacDiarmid Institute for Advanced Materials and Nanotechnology, School of Physical and Chemical Sciences, Te Kura Matū, University of Canterbury, Christchurch, New Zealand; The MacDiarmid Institute for Advanced Materials and Nanotechnology, School of Physical and Chemical Sciences, Te Kura Matū, University of Canterbury, Christchurch, New Zealand; Electrical and Electronics Engineering, University of Canterbury, Christchurch, New Zealand; School of Mathematical and Physical Sciences, University of Technology Sydney, Australia; The MacDiarmid Institute for Advanced Materials and Nanotechnology, School of Physical and Chemical Sciences, Te Kura Matū, University of Canterbury, Christchurch, New Zealand

**Keywords:** Complex network, Scale-free topology, Scale-free dynamics, Long-range temporal correlations, Nanoparticle network, Neuromorphic computing

## Abstract

Biological neuronal networks are the computing engines of the mammalian brain. These networks exhibit structural characteristics such as hierarchical architectures, small-world attributes, and scale-free topologies, providing the basis for the emergence of rich temporal characteristics such as scale-free dynamics and long-range temporal correlations. Devices that have both the topological and the temporal features of a neuronal network would be a significant step toward constructing a neuromorphic system that can emulate the computational ability and energy efficiency of the human brain. Here we use numerical simulations to show that percolating networks of nanoparticles exhibit structural properties that are reminiscent of biological neuronal networks, and then show experimentally that stimulation of percolating networks by an external voltage stimulus produces temporal dynamics that are self-similar, follow power-law scaling, and exhibit long-range temporal correlations. These results are expected to have important implications for the development of neuromorphic devices, especially for those based on the concept of reservoir computing.

## Author Summary

Biological neuronal networks exhibit well-defined properties such as hierarchical structures and scale-free topologies, as well as a high degree of local clustering and short path lengths between nodes. These structural properties are intimately connected to the observed long-range temporal correlations in the network dynamics. Fabrication of artificial networks with similar structural properties would facilitate brain-like (“neuromorphic”) computing. Here we show experimentally that percolating networks of nanoparticles exhibit similar long-range temporal correlations to those of biological neuronal networks and use simulations to demonstrate that the dynamics arise from an underlying scale-free network architecture. We discuss similarities between the biological and percolating systems and highlight the potential for the percolating networks to be used in neuromorphic computing applications.

## INTRODUCTION

Next-generation computing concepts like big data, artificial intelligence, and the internet of things demand high parallelism, error tolerance, and more energy-efficient platforms (Cheng, Ríos, Pernice, Wright, & Bhaskaran, 2017). Current computing architectures based on digital logic cannot keep up with the demand as they are hampered by the inability to continually scale complementary metal-oxide-semiconductor technology and by the von Neumann bottleneck (Markov, 2014). Inspired by the human brain, neuromorphic computing is a novel computing paradigm that seeks to overcome such limitations, and that is attracting attention for its potential to enable complex computing tasks such as associative memory (Hu et al., 2015), pattern detection (Wang et al., 2018), and classification (Prezioso et al., 2015). A wide range of components have been explored for neuromorphic hardware platforms including artificial silicon-based neurons (Mahowald & Douglas, 1991), cross-bar memristors (Prezioso et al., 2015; Wang et al., 2018), and novel nanoscale elements that mimic the behavior of synapses and neurons (Terabe, Hasegawa, Nakayama, & Aono, 2005; Tuma, Pantazi, Le Gallo, Sebastian, & Eleftheriou, 2016). These approaches aim to emulate the functionality of both neurons and synapses at the individual level, while large-scale neuromorphic computing systems (Davies et al., 2018; Furber, 2016; Hopkins, Pineda-García, Bogdan, & Furber, 2018; Merolla et al., 2014) are built by utilizing regular arrays of these elements.

Biological neuronal networks on the other hand are intrinsically complex and operate through a massive number of nonlinearly interacting neurons (Bullmore & Sporns, 2012). Biological networks show emergent phenomena (Chialvo, 2010), that is, the networks exhibit behavior that cannot be produced by the individual elements in isolation. Recent studies have shown that neuronal networks have small-world properties (Bullmore & Sporns, 2009, 2012; Park & Friston, 2013) and are hierarchically organized (Barabási & Oltvai, 2004; Park & Friston, 2013) with fractal geometry (Di Ieva, Grizzi, Jelinek, Pellionisz, & Losa, 2014; Werner, 2010), and that the underlying network has a scale-free topology (Bonifazi et al., 2009; Eguíluz, Chialvo, Cecchi, Baliki, & Apkarian, 2005). These network features bestow the brain with a number of functional benefits such as the ability to efficiently transport energy, minimal wiring cost, and maximum local autonomy along with global connectivity (Bullmore & Sporns, 2012; Park & Friston, 2013).

Inspired by structural characteristics of biological neuronal systems, scale-free, hierarchical networks are predicted to enable neuromorphic computing approaches such as reservoir computing (RC) (Lukoševičius & Jaeger, 2009; Maass, Natschläger, & Markram, 2002). A reservoir is a complex network of highly interconnected nonlinear nodes that evolve dynamically in response to input signals. The reservoir maps input signals onto outputs of higher dimensions, which are then examined by an external readout function (Lukoševičius & Jaeger, 2009). The reservoir computing concept has been discussed extensively in recent literature (see for example Du et al., 2017 and Riou et al., 2019, and recent reviews by Dale, Stepney, Miller, & Trefzer, 2016 and Tanaka et al., 2019) and the concept is further illustrated in the Supporting Information: Figure 1. While simulations of scale-free reservoirs in software show significant performance enhancement over conventional random reservoirs (Deng & Zhang, 2007), such reservoirs are yet to be implemented experimentally.

The information processing in the brain is spatiotemporal in nature (Saha et al., 2013): it relies not only on underlying *structure* but also on collective neuronal *dynamics* over time. Indeed, recent electrophysiological studies show that the fluctuations of collective neuronal activity exhibit rich temporal properties, including scale-free and self-similar temporal dynamics and long-range temporal correlations (LRTC; Linkenkaer-Hansen, Nikouline, Palva, & Ilmoniemi, 2001; Palva et al., 2013). The presence of LRTC in the dynamics of neuronal populations indicates that activity at any given time is being influenced by the history of the system, which is essential for decision-making and working-memory tasks (Meisel, Bailey, Achermann, & Plenz, 2017). LRTC in neuronal dynamics has also been linked to efficiency in learning, memory formation, rapid information transfer, and network reorganization (Botcharova, Farmer, & Berthouze, 2014). The link between LRTC and information processing is further supported by the insight that LRTCs are generic features of a critical point: the positioning of a dynamical system at a phase transition between two qualitatively different types of states where both spatial and temporal correlations emerge (Bak & Chen, 1989; Watkins, Pruessner, Chapman, Crosby, & Jensen, 2016). Numerous computational and experimental studies have provided support for the hypothesis that the brain also operates at or near a critical point (Beggs & Plenz, 2003; N. Friedman et al., 2012) and thereby exploits the computational advantages provided by criticality (Muñoz, 2018). Temporal correlation is an essential aspect of neuromorphic computing (Stieg et al., 2012; Torrejon et al., 2017) and is a prerequisite for RC approaches such as the echo state network (Lukoševičius & Jaeger, 2009). The realization of devices with the topological and dynamical features of biological neuronal networks would open new avenues for neuromorphic computing, particularly RC (Muñoz, 2018; Srinivasa, Stepp, & Cruz-Albrecht, 2015). Note however that the aim of neuromorphic computing is to enable new ways of performing computational tasks, such as pattern recognition, that are performed very efficiently by the brain. The aim is to exploit examples of brain-like structures, functionality, and/or dynamics, rather than to attempt to replicate the enormous complexity of the brain in every detail.

In the present study, we focus on self-organised networks of metallic nanoparticles, poised at the phase transition between an insulating phase and a conducting phase (Bose, Mallinson, Gazoni, & Brown, 2017; Bose, Shirai, Mallinson, & Brown, 2019; Mallinson et al., 2019; Sattar, Fostner, & Brown, 2013). Our experiments and numerical simulations show that the underlying *networks* have scale-free topology, hierarchical architectures, and small-world attributes such as a high degree of local clustering and a short average path length. When activated by external stimuli, this topology ensures that the resulting network *dynamics* are also self-similar, scale-free, and show LRTC. We compare the network topology and dynamics to those of biological neuronal networks, and demonstrate strong similarities. These results take us a step closer to realizing neuromorphic computing devices with brain-like spatiotemporal properties.

## RESULTS

### Physical Network Structure

**Figure 1.**
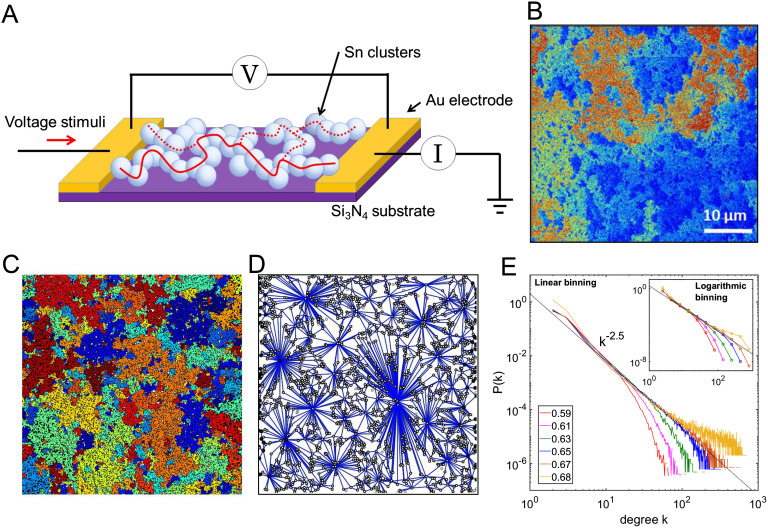
Percolating nanoparticle networks have fractal geometries and scale-free topology. (A) Schematic illustration of our two-terminal device geometry comprising a percolating nanoparticle network. The solid line represents an Ohmic conduction pathway at *p* ∼ *p*_*c*_, while the dotted lines represent tunneling pathways in the network. (B) The postdeposition colorized scanning electron micrograph of a device indicating that the morphology of the nanoparticle network has fractal geometry (scale bar = 10 *μ*m). Colorization enhances visualization of connected groups of particles. (C) Numerical simulation of a system size of 200 × 200 particle diameters near the percolation threshold showing groups of particles (represented by different colors) that are separated by tunnel gaps. (D) The map of connections between different groups of particles (nodes) in (C) exhibits a highly inhomogeneous degree distribution. (E) The probability density function (PDF) of the degree distribution at different surface coverages. The data are displayed using linear bins, while the insert shows the same distribution with logarithmic bins in order to demonstrate that the heavy-tailed distribution is independent of the binning method. Each color represents a specific surface coverage, while the black dotted line is a guide for the eye corresponding to an exponent of 2.5. The degree distribution near percolation threshold (blue and orange lines) is heavy-tailed and is close to a power-law with exponent 2.5, suggesting that the underlying network has scale-free topology.

Our percolating networks of nanoparticles are formed through simple deposition techniques: A substrate with preprepared electrodes is placed in a beam of particles from a magnetron sputtering source (Bose et al., 2017; Sattar et al., 2013). When deposited on the substrate (see Materials and Methods), particles come into contact and form interconnected groups that are separated by tunnel gaps (Supporting Information: Figure 2). The state of the system is controlled by the surface coverage *p* (Supporting Information: Figure 3). At the percolation threshold *p* ∼ *p*_*c*_ (∼ 68%), the system undergoes a second-order phase transition and the deposition is terminated at this critical point. The resultant devices are self-organized in the sense that the key structures result from the statistical properties of the randomly deposited percolating networks and the intrinsic physical processes that take place when the particles land on the chip (Bose et al., 2017; Sattar et al., 2013), and no manipulation of the deposited component is required. Figure 1A shows a schematic diagram of our two-electrode device, comprising groups of conducting nanoparticles deposited at the percolation threshold. The postdeposition colorized scanning electron micrograph (SEM) image in Figure 1B shows that the groups have varying sizes and fractal geometries, consistent with standard percolation theory (Voss, Laibowitz, & Allessandrini, 1982; Stauffer & Aharony, 2003). The tunnel gaps between groups can act as switching sites: Upon application of an external voltage stimulus, atomic-scale filaments can be formed (and subsequently broken) in the tunnel gaps, resulting in changes in the network conductance (Sattar et al., 2013).

These percolating tunneling networks have been the subject of relatively limited study (Fostner, Brown, Carr, & Brown, 2014; Fostner & Brown, 2015; Grimaldi, 2014). Their structural properties are difficult to analyze in the experimental system because of the conflicting requirements to analyze large areas and at the same time to resolve atomic-scale details. Hence, we begin by simulating the percolating tunneling network for various surface coverage (see the Supporting Information and refs. Fostner et al., 2014, and Fostner & Brown, 2015, for details of the simulations). An example of a simulated network near the percolation threshold is shown in Figure 1C, where connected groups of particles are labeled with a single color. Figure 1D presents a map of the connections between these groups. Note that the conduction through the system is dominated by the tunnel gaps that are represented by the blue lines in Figure 1D (and as illustrated schematically in the Supporting Information: Figure 2). It is immediately apparent that the number of connections *k* between one group and another is highly inhomogeneous, with some groups much more connected than others. This inhomogeneity increases as the system approaches the percolation threshold, where critical fluctuations become important (see the Supporting Information: Figure 4; Stauffer & Aharony, 2003). Figure 1E shows the degree distribution, *P*(*k*), which is a fundamental characteristic of the network (Albert & Barabási, 2002; Strogatz, 2001), for different coverages. At low network coverages (0.59 and 0.61), the tails of the degree distribution have an exponential cutoff (these are not finite size effects—see the Supporting Information: Figure 5). Near the percolation threshold (coverage of 0.65 to 0.67) the degree distribution is heavy-tailed and is close to a power-law *P*(*k*) ∼ *k*^−*φ*^ with *φ* ∼ 2.5. For many complex networks, such as biological neuronal networks, *φ* was reported to be between 2.0 to 3.0 (Barabási & Oltvai, 2004). Hence, our percolating networks have similar connectivity to biological networks and are scale-free (Strogatz, 2001). Importantly, the power-law degree distribution ensures that there is a small number of heavily connected nodes that promote global connections across the system (Park & Friston, 2013). We emphasize that this scale-free behavior results from the geometry of the tunneling connections between groups of particles. These tunnel gaps are not considered in standard forms of percolation theory, where for *p* > *p*_*c*_ conduction takes place along a fractal backbone (Voss, 1984) and where for *p* < *p*_*c*_ there is no conduction between the fractal-shaped groups (Voss et al., 1982). It is worth noting here that further important quantities such as branching ratios (Beggs & Plenz, 2003), percolation critical exponents (Stauffer & Aharony, 2003), and precise universality class (Sethna, Dahmen, & Myers, 2001) are yet to be elucidated for percolating-tunneling systms.

Further analysis (Supporting Information: Figure 5B, C) shows that our percolating networks have a high clustering coefficient and a low average path length, which is indicative of a small-world topology (Bullmore & Sporns, 2009, 2012; Strogatz, 2001). More detailed analysis indicates that our networks have hierarchical architectures (Supporting Information: Figure 5D) hat seamlessly integrate a scale-free topology with a high degree of local clustering (Barabási & Oltvai, 2004). These are the same network features that provide the structural basis for stability and diversity of functional patterns in cortical networks (Kaiser, 2007), and for high efficiency of information transfer in the brain (Bullmore & Sporns, 2009, 2012). Hierarchical modularity also plays a major role in regulating the dynamics of neuronal networks that are more complex in modular networks than corresponding random networks (Sporns, Tononi, & Kötter, 2005). Hierarchical modular networks assist metastability (Wildie & Shanahan, 2012) and they are an important structural constituent for enabling the dynamic regime of criticality (Rubinov & Sporns, 2010), which is characterized by spontaneous and persistent bursts of activity. Simulations have also shown that hierarchical network structure and critical dynamics are mutually reinforcing (E. J. Friedman & Landsberg, 2013). Finally, clustered and hierarchical topologies are particularly suited to local (segregated) operation and to the global integration of segregated functions (Park & Friston, 2013), and ensure that the brain has high network efficiency at low energy and connection cost (Bullmore & Sporns, 2012).

### Burstiness and Distributed Switching Activity

**Figure 2.**
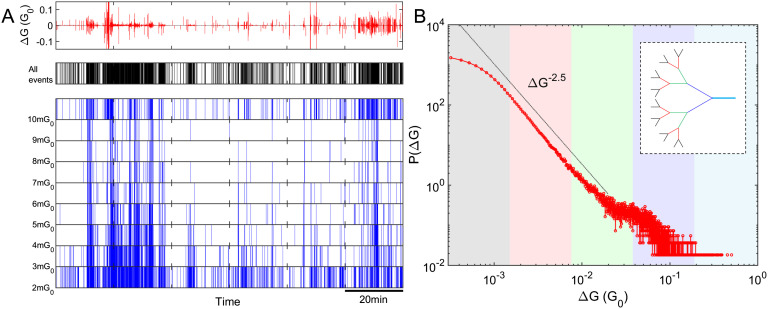
Percolating nanoparticle networks produce complex patterns of switching events that are bursty in nature and are distributed across the fractal network. (A) (Top panel) The vertical scale shows change in conductance Δ*G* (units of *G*_0_ = 2*e*^2^/*h*, the quantum of conductance, are used for convenience; Sattar et al., 2013). (Middle panel) A typical example of a switching activity sequence (event-train) exhibiting bursty dynamics, that is, significantly enhanced activity levels over short periods of time followed by periods of inactivity. (Lower panel) The event-train plot from the top panel is plotted in multiple ranges of Δ*G*. The bursty subsections contain events with a wide range of Δ*G*, pointing to a nonlocal origin of events within bursts. (B) The probability density function (PDF) of changes in total network conductance, *P*(Δ*G*), resulting from switching activity is heavy-tailed. The inset shows a schematic illustration of a highly branched fractal network. The *P*(Δ*G*) distribution is divided into segments represented by various color shades, where smaller Δ*G* (e.g., pink-gray shaded regions) generally originates from higher order branches (e.g., red or black branches in the inset), while larger Δ*G* (e.g., blue shaded region) mostly originates from lower order branches (e.g., blue branch in the inset). The apparent deviation in the tail of the distribution is most likely due to insignificant statistical fluctuations.

Under application of external voltage stimuli, our devices exhibit discrete changes in conductance (Δ*G*), which are termed *switching events*. As mentioned above, each switching event is caused by formation or destruction of an atomic-scale filament in a tunnel gap (Sattar et al., 2013). The top panel of Figure 2A shows a typical example of temporal evolution of Δ*G*. The sequence of switching events can be represented as an *event-train* (Karsai, Kaski, Barabási, & Kertész, 2012), that is, a time series with a value of 1 if an event takes place (Δ*G* is above the detection threshold (Supporting Information: Figure 7), otherwise a value of 0, and is qualitatively similar to sequences of action potentials from biological neurons in the brain (Matzner & Bar-Gad, 2015). A typical event-train is shown in the middle panel of Figure 2A. We observe highly inhomogeneous bursty switching activity (Karsai et al., 2012), characterized by periods of intense activity followed by periods of quiescence. Such bursty activity has been reported in many natural phenomena including earthquakes, solar flares, and neuronal activity (Karsai et al., 2012). In order to illustrate the burstiness of the data, we generate event-train plots for multiple ranges of Δ*G* as shown in the lower part of Figure 2A. The bursty subsections contain a wide range of Δ*G* values, indicating that switching is not local behavior on one branch of the network, but is instead a consequence of correlated activity that is distributed across the network.

Figure 2B shows the distribution of event sizes, *P*(Δ*G*), and the inset is a schematic representation of a fractal branched conduction pathway. While each atomic filament has the same conductance (Sattar et al., 2013), their contribution to the change in network conductance can vary over several orders of magnitude depending on the configuration of the network and the location of the switching site within the network. Switches on “main branches” (which have few parallel pathways, e.g., the blue branch in the inset) cause larger changes in network conductance (blue shaded region in Figure 2B). Conversely, atomic switches on “higher order” branches (which have many parallel current pathways, e.g., the red or black branches in the inset) cause a smaller Δ*G* (pink-gray shaded regions in Figure 2B). Switching events that have similar Δ*G* may be the result of either repeated activity at a single site, or activity at different sites that sit on different branches of similar order. Note that when an atomic switch changes state it reconfigures the conduction pathways through the network, and so a switching site may find itself located on a different branch following the occurrence of a switching event at another location. *P*(Δ*G*) spans ∼3.5 orders in Δ*G* and is heavy-tailed (the guide line on Figure 2B indicates a power-law exponent of 2.5), supporting the assertion that the switching activity is highly distributed throughout the different branches of a fractal network. It is worth noting that the distribution of event sizes in biological neuronal networks is not accessible for comparison because of relatively low signal-to-noise ratio in those experiments (Gireesh & Plenz, 2008; Shew et al., 2015).

### Scale-Free and Correlated Temporal Dynamics

**Figure 3.**
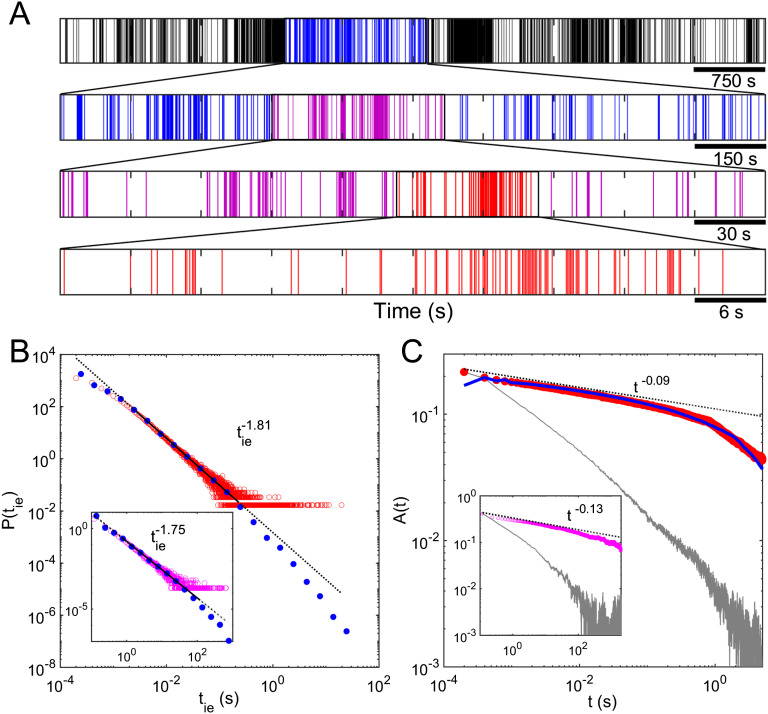
The temporal dynamics of nanoparticle networks are self-similar and exhibit power-law scaling behavior and long-range temporal correlation (LRTC). (A) The switching activity patterns are not readily distinguishable at various levels of temporal magnification, which suggests the presence of self–similar temporal dynamics. (B) The PDF of interevent intervals (IEIs) between successive switching events follows a power-law distribution, suggesting scale-free dynamics. Red: distribution presented using standard (linear) bin sizes; blue: distribution presented using logarithmic bin sizes to allow visualization of the heavy tail; the fitting line is represented by the black line, which is solid over the fitting range and dashed otherwise. (C) The autocorrelation function (ACF) has slow power-law decay over several orders (red), suggesting existence of LRTC in switching dynamics. Random shuffling of the IEI sequence results in significant increase in ACF decay slope (gray), indicating that the original data are highly correlated. The ACF plot represented by the blue line was obtained using a model that implements correlation between IEIs drawn from a power-law distribution (see the Supporting Information). The resulting ACF slope is similar to the experimental ACF slope (red line), implying that the experimental IEI sequence is highly correlated. The data shown in the insets of (B) and (C) are obtained for the same device at identical applied voltage but using a different measurement system with the sampling rate 1,000 times lower. The resulting slopes are almost identical irrespective of sampling rate, which gives further evidence of temporal self-similarity.

To visualize the switching patterns on different temporal scales, we generated event-train plots for multiple timescales; a typical example is shown in Figure 3A. The top panel shows a section of signal comprising 7,500 s of data, while the subsequent panels show sections of the top panel with the temporal scale magnified 5, 25, and 125 times. The switching activity patterns in the four panels are not readily distinguishable, which indicates that the switching events on different timescales are qualitatively self-similar. This self-similarity is independent of sampling rate (see the Supporting Information: Figure 6 for details), and hence is an intrinsic property of our devices. Self-similar temporal dynamics are typical in electrophysiological signals from the cortex (Werner, 2010), and biological physiological measurements such as temporal patterns of heartbeats (Goldberger et al., 2002) have been interpreted as a consequence of an underlying fractal physical structure (Di Ieva et al., 2014; Werner, 2010) and are a signature of correlations on many temporal scales (Linkenkaer-Hansen et al., 2001).

To quantify the temporal structure of switching events, we investigated the interevent interval (IEI) distribution (Segev et al., 2002). The IEIs (*t*_*ie*_) are defined as the time between two consecutive events. Figure 3B shows that the IEI distribution follows a power-law *P*(*t*_*ie*_) ∼ $t_{ie}^{-\gamma }$ with an exponent *γ* ∼ 1.8, implying scale-free dynamics. The fit is based on the maximum likelihood (ML) approach and the Kolmogorov-Smirnov (KS) test (see Materials and Methods). The inset of Figure 3B shows the IEI distribution of the same device at identical applied voltage, but using a different measurement system with 1,000 times slower sampling rate (see Materials and Methods) than that used in Figure 3B, yet the power-law exponent obtained is almost identical (*γ* ∼ 1.75), which gives strong evidence of scale-free temporal dynamics. These results are consistent with previously reported heavy-tails in the interspike interval distributions of neuronal populations of in vitro cortical cultures that have been interpreted as a feature of an excitable system consisting of many nonlinearly interacting subsystems (neurons; Segev et al., 2002).

A power-law IEI distribution alone does not prove correlation among switching events (Karsai et al., 2012; Vajna, Tóth, & Kertész, 2013). Therefore, in order to characterize the correlation between switching events, the autocorrelation function (ACF) was calculated. The ACF is a measure of the correlation between the observed signal and a delayed version of itself. The correlation strength can be inferred from the amplitude of the ACF (Meisel et al., 2017; Scheffer et al., 2009), while persistence of temporal correlation can be inferred from the slope of the ACF plot (Meisel et al., 2017). Figure 3C presents our experimental ACF for both fast and slow (inset) sampling rates. Both ACF plots show a power-law decay for more than 3.5 orders of magnitude in time with almost identical decay slope (*β* ∼ 0.1), which further consolidates the evidence for self-similarity in temporal dynamics and suggests the existence of LRTCs (Linkenkaer-Hansen et al., 2001; Palva et al., 2013). To verify this conclusion, randomized time series of switching events were obtained by shuffling the original IEIs. This approach preserves the total number of switching events and the IEI distribution, but removes temporal correlations between the switching events. The ACF plot obtained from shuffled IEIs (gray line) decays much faster than our experimental ACF plots (Vajna et al., 2013), confirming LRTC in the original switching sequences. These temporal characteristics are invariant across devices (Supporting Information: Figure 8), showing that they are intrinsic properties of the network. In neuroscience it is usually difficult to directly measure ACFs and so detrended fluctuation analysis is often the preferred method for quantifying LRTC (see refs. Palva et al., 2013; Meisel et al., 2017, and citations therein). To facilitate a comparison we use the relationship *β* = 2 − 2*H* (Meisel et al., 2017) to estimate the Hurst exponent (*H*) and find *H* ∼ 0.9, confirming the strong correlations in percolating tunneling systems.

The power-law decay in *P*(*t*_*ie*_) and ACF show that the distribution of switching events *lack a characteristic time* (Papo, 2013). The presence of LRTC suggests that each burst is only a part of a series of correlated events, and the fractal temporal structure (as shown in Figure 3A) reflects a hierarchy of bursts within bursts (Linkenkaer-Hansen et al., 2001). In order to demonstrate that LRTC in our device is due to a hierarchy of bursts, we have modeled the relationship between the ACF and the type of burstiness (see the Supporting Information: Figure 9). In this model we take uncorrelated but power-law-distributed IEIs as input and then we introduce correlation between successive IEIs by organizing them hierarchically. We then calculate the ACF and compare with experimental ACF plots. A typical result is presented in Figure 3C, where the ACF obtained from the model (blue line) has a similar decay slope to the experimental ACF (red line). As shown in the Supporting Information, neither nonbursty event-trains nor bursty event-trains that lack a hierarchy exhibit the power-law slope observed experimentally. The essential point is that hierarchical bursting must be included in the model in order to reproduce the experimental crrelations. This strongly supports the conclusions that our events are highly correlated and that the correlation stems from their inherent hierarchical organization.

### Voltage Dependence of the Temporal Switching Activity

**Figure 4.**
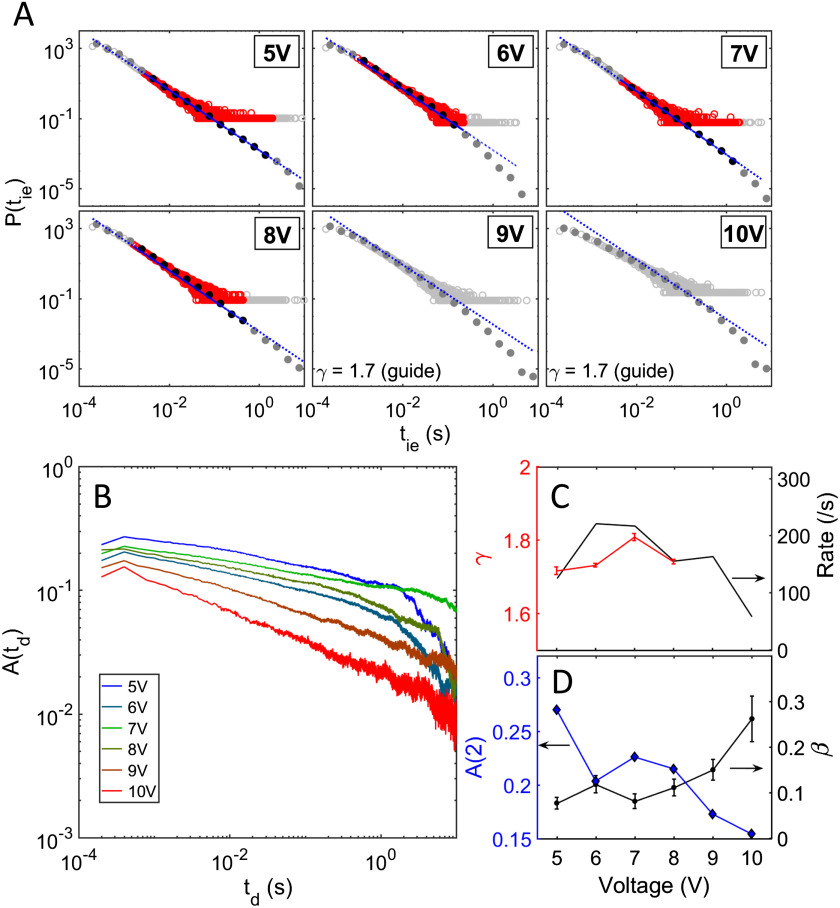
Evolution of switching dynamics and correlation strength with applied voltage. (A) Distribution of interevent intervals (IEIs) for a range of applied DC voltages between 5 V and 10 V. For 5–8 V the data are consistent with a power-law with slope ∼1.7, but at higher voltages (9 V and 10 V) the data do not follow a strict power-law (see Materials and Methods for details of the fitting procedure). (B) Corresponding autocorrelation functions (ACFs). (C) Variation of IEI exponent (*γ*) and switching rate as a function of applied voltage. (D) Dependence on applied voltage of the ACF decay exponent (*β*) and lag-2 autocorrelation amplitude at second time delay, A(2). The maximum correlation strength is observed at lower operating voltages. At voltages higher than 8 V, the slope of the ACF increases while A(2) decreases, implying a decrease in correlation. While there is also a decrease in the switching rate at higher voltages (see C), a decrease in activity is generally associated with an *increase* in the correlation strength (Scheffer et al., 2009). Therefore the decrease in correlation at higher voltage cannot be attributed to the lower switching rate.

In order to investigate the stimulus dependence of the scale-free network activity and temporal correlation, a range of DC voltages between 5 V (slightly above the switching threshold voltage) and 10 V were applied to our devices. Figure 4A shows IEI distributions, while Figure 4B shows ACFs for the range of applied stimuli. The IEI distributions are heavy-tailed, spanning 4 orders of magnitude in time for all six voltages, while the ACFs for all voltages exhibit LRTC with decay for over ∼4 orders of magnitude in time. To quantify the voltage dependence of the ACFs, the correlation strength (the ACF at lag-2; see Materials and Methods) and the slope of the ACF were calculated and are depicted in Figure 4D. We applied the ML approach and the KS test to validate the power-law hypothesis in *P*(*t*_*ie*_). For lower voltages (5–8 V) power-law fits to *P*(*t*_*ie*_) pass the KS test over more than 2 orders of magnitude in time, yielding the slopes shown in Figure 4C. The lag-2 correlation and the ACF slope also vary only weakly, suggesting that the correlation strength is independent of the applied voltage up to 8 V. Interestingly, at higher voltages (9–10 V) *P*(*t*_*ie*_) deviates from a strict power-law (Figure 4A), the correlation strength at lag-2 is significantly lower than that at 5–8 V, and the ACF slope is much higher. Hence there is a clear decrease in correlation at voltages higher than 8 V.

In the neuroscience literature, a decline in LRTC has been linked to neurological conditions such as Alzheimer’s disease (Montez et al., 2009) and schizophrenia (Nikulin, Jönsson, & Brismar, 2012), in addition to a number of cognitive alterations including sustained-wakefulness (Meisel et al., 2017) and focused attention meditation (Irrmischer et al., 2018). In the case of Alzheimer’s disease (Montez et al., 2009), the decrease in correlation is attributed to a disconnection in large-scale networks, while in the case of sustained-wakefulness, the decrease in correlation is attributed to fatigue in the brain because of sleep deprivation (Meisel et al., 2017). In our device, one may speculate that a similar fatiguing process could be responsible for the decline in LRTC observed at 9–10 V: Higher applied voltage results in larger currents that may well cause deformation at switching sites because of excessive Joule heating. As such large currents are distributed across the network, there may be network-wide fatigue, causing disconnections between different parts of the network. This would disrupt the propagation of local interaction to global scale (Linkenkaer-Hansen et al., 2001) leading to the observed decrease in correlation. Nonetheless, the network dynamics of our devices show maximum correlation at low voltages, which would be the operating point of the devices.

## DISCUSSION

Biological neuronal networks generally exhibit small-world properties such as a high degree of local clustering and a short average path length between all node pairs, as well as hierarchical network structures and scale-free topologies (Bonifazi et al., 2009; Bullmore & Sporns, 2012; Eguíluz et al., 2005; Park & Friston, 2013). These network properties are important to the functionality of the brain, including efficient information processing, adaptability, and divergent functionality within a fixed structure (Park & Friston, 2013), as well as to the observed dynamics (van den Heuvel & Hulshoff Pol, 2010). For example, hierarchical or fractal network topologies confer robustness to dynamics when the connections between nodes are reconfigured (Robinson, Henderson, Matar, Riley, & Gray, 2009), and lead to the emergence of scale-free temporal dynamics and LRTC (Bullmore & Sporns, 2012; Werner, 2010; Zhigalov, Arnulfo, Nobili, Palva, & Palva, 2017). Furthermore, hierarchical modular structures can lead to an extended parameter range for critical behavior known as the Griffiths phase, which is associated with a generic enhancement of functionality (Moretti & Muñoz, 2013; Zhigalov et al., 2017).

The relationships between spatial and temporal correlations, connections to concepts of self-organized criticality, and the diversity of systems in which they are observed have been discussed extensively in the literature (see Cavagna, Giardina, & Grigera, 2018 and Watkins et al., 2016, for reviews) but are still subjects of intense investigation (Chialvo, Cannas, Plenz, & Grigera, 2019). In systems near criticality, long-range spatial and temporal correlations are different sides of the same coin, that is, they are interdependent (Bak & Chen, 1989). Long-range spatial correlations ensure that local changes can propagate throughout the network on multiple timescales, leading to LRTC (Linkenkaer-Hansen et al., 2001). Long-range spatial correlations are inherent to our system, as a consequence of deposition at the percolation threshold, where the correlation length diverges (Stauffer & Aharony, 2003). Like the brain, our devices consist of a network of nonlinearly interacting nodes, and temporal correlations and fractal dynamics emerge from the underlying fractal structure and local processes that connect the nodes. It should be noted, however, that in biological systems even single cells can exhibit fractal dynamics (Johnson, Wright, Xiá, & Wessel, 2019). In the brain, synapses connect/transmit information between neurons. In our networks, switching events occur in tunnel gaps by formation and annihilation of atomic-scale wires (Bose et al., 2017, 2019; Sattar et al., 2013). Under the application of external DC bias, the formation (or annihilation) of an atomic wire at a tunnel gap redistributes current across the entire network, thereby modifying local electrostatic potentials across other tunnel gaps, and leading to bursts of switching events, which are similar to neuronal avalanches (Beggs & Plenz, 2003; N. Friedman et al., 2012; Mallinson et al., 2019). In other words, each switching event influences subsequent switching events through internal feedforward and feedback networks, giving rise to temporal correlations.

The observation of LRTC in neuronal networks has several implications for information processing: (a) LRTCs have been suggested to reflect the degree to which the brain remains capable of quick reorganization (Linkenkaer-Hansen et al., 2001), providing responsiveness to different processing demands. (b) Information processing and learning in the human brain are believed to be carried out by correlated firing patterns of neuronal populations (Franke et al., 2016). (c) LRTCs are associated with extended timescales and memory effects, which are thought to provide favorable neuronal substrates for the integration of information across time and across different cortical areas in order to increase the signal-to-noise ratio during cognitive tasks (e.g., during decision-making; Meisel et al., 2017). (d) A network consisting of many subunits (such as neurons) that shows LRTC may provide a distributed network for memory (Bhattacharya, Edwards, Mamelak, & Schuman, 2005). (e) The presence of LRTC in cortical dynamics is consistent with the idea that the brain may be operating in a self-organized critical state (Bhattacharya et al., 2005; Chialvo, 2010; Linkenkaer-Hansen et al., 2001; Palva et al., 2013), where dynamic range and memory are maximized—both favorable features for information processing (Palva et al., 2013).

The correlated network dynamics discussed above are consistent with criticality in our percolating networks (Mallinson et al., 2019) and might be exploited within the context of a practically implementable neuromorphic computational framework such as RC (Lukoševičius & Jaeger, 2009; Stieg et al., 2012). Software simulations of RC have acknowledged the importance of utilizing the rich topological features of biological neuronal networks; for example, Deng and Zhang (2007) simulated the use of a scale-free and highly clustered reservoir for RC, leading to improved performance in a chaotic time series prediction task. Similarly, reservoirs with a high degree of clustering have shown better performance in memory capacity tasks as compared with a random reservoir (Rodriguez, Izquierdo, & Ahn, 2019), while hierarchical structured reservoirs are particularly suitable to process time series with multiscale characteristics such as speech, text, and gestures (Lukoševičius & Jaeger, 2009). To date a reliance on lithographic processing for fabrication of neuromorphic hardware has meant that these network features of the brain have typically been ignored in favor of highly regular architectures (Furber, 2016; Merolla et al., 2014; Prezioso et al., 2015; Wang et al., 2018). Our self-organised devices, which have several topological features of biological neuronal networks and exhibit scale-free and self-similar temporal dynamics, and LRTC, therefore provide an interesting alternative platform for bio-realistic neuromorphic hardware implementation.

To summarize, our networks of nanoparticles operate near a critical point (the percolation phase transition) and exhibit structural features including scale-free topology and hierarchical architectures that are similar to those of the brain. These structural features therefore lead to LRTCs that are similar to those of biological systems. Spatial and temporal correlations are also key requirements for reservoir computing systems. Given the similarities between the structural and temporal dynamics of biological neuronal networks, we propose that our percolating nanoparticle networks provide an ideal physical system for implementation of hardware-based reservoir computing.

## MATERIALS AND METHODS

### Device Fabrication

Our percolating devices are fabricated by simple nanoparticle deposition processes (Bose et al., 2017; Sattar et al., 2013; Schmelzer, Brown, Wurl, Hyslop, & Blaikie, 2002). Sn nanoparticles of 7 nm are deposited between gold electrodes (spacing 100 *μ*m) on a silicon nitride surface and coalesce to form particles of 20 nm diameter. Deposition is terminated at the onset of conduction, which corresponds to the percolation threshold (Schmelzer et al., 2002; Stauffer & Aharony, 2003). The deposition takes place in a controlled environment with a well-defined partial pressure of air and humidity, as described in Bose et al. (2017). This process leads to controlled coalescence and fabrication of robust structures that function for many months, but that yet allow atomic-scale switching processes to take place unhindered.

### Electrical Stimulus and Measurement

Electrical stimuli are applied to the electrode on one side of the percolating device, while the opposite electrode of the system is held at ground potential. While a variety of types of stimuli (voltage pulses, ramps) can be applied to the system, constant DC voltages are used in this work because they facilitate observation of ongoing reconfigurations of the states of the switches in the devices. Measurements over long time periods are necessary to avoid significant cutoffs in the power-law distributions (Clauset, Shalizi, & Newman, 2009; Deluca and Corral, 2013). Here we present data from DC stimulus of four devices (see Figure 3 and Supporting Information: Figure 8), but the data presented are consistent with that obtained from DC, pulsed, and ramped voltage stimulus of a further 10 devices.

Our electrical measurements are performed using two distinct sets of measurement electronics, to allow measurement of the device conductance on two distinct timescales. The first method relies on a picoammeter and is limited to a relatively slow sampling rate (0.1 s sampling interval). The second method utilizes a digital oscilloscope allowing a much higher sampling rate (200 *μ*s sampling interval for the data presented here). As shown in Figure 3, both methods resulted in qualitatively and quantitatively similar data, with similar power-law exponents for each of the main quantities of interest. Our results and conclusions are therefore not influenced by the sampling rate.

### Data Analysis

The data analysis methods used in this work are substantially the same as those developed in the neuroscience community to analyze micro-electrode array recordings from biological brain tissue. Events are defined as changes in the conductance signal that exceed a threshold value (Supporting Information: Figure 7). We show in Supporting Information Figure 7 that, as in the neuroscience literature, the choice of threshold in a reasonable range does not significantly affect the IEI distributions and *A*(*t*). Note that, since lag-1 of ACF may be affected by finite sampling rate, we adopt lag-2 as an indicator of the correlation strength.

### Fitting and Goodness-of-Fit

We follow the maximum likelihood (ML) approach of Clauset et al. (2009) and Deluca and Corral (2013) to estimate power-law exponents in the IEI distributions. The ML estimators are obtained for both power-law and exponential distributions.

We use the Akaike information criterion (Wagenmakers & Farrell, 2004) to identify which distribution is more likely and find in all cases that it is the power-law. We then generate 500 random power-law and exponential distributions using the calculated ML estimators, and iterate the choice of cutoffs (*t*_min_ and *t*_max_) for the data range, finding the probability of obtaining a Kolmogorov-Smirnov (KS) distance (Deluca and Corral, 2013) at least as the data from the device. In all cases, we fail to reject the null hypothesis that IEI distributions are power-law-distributed (we require *p* values > 0.2), but we do reject the null hypothesis that the distributions are exponentially distributed (we find *p* values < 0.01; Supporting Information: Figure 10).

The KS test is extremely sensitive to small deviations from a mathematical power-law (Deluca & Corral, 2013; Marshall et al., 2016). The deviation from power-law behavior in our data is rather small (see Figure 3), but we nevertheless follow Deluca and Corral (2013) and Marshall et al. (2016) and allow the data to be truncated in order to show definitively that the distributions are power-law. The IEI distributions are found to be power-law over about 2 orders of magnitude in time, with both a lower and an upper cutoff (see more details in the Supporting Information: Figure 11).

The ML methods cannot be applied to data which is not in the form of a probability distribution, and so the standard linear regression techniques are used to obtain the measured exponents for *A*(*t*).

## ACKNOWLEDGMENTS

The authors acknowledge useful discussions with P. Bones, J. Beggs, C. Young, and N. McNaughton.

## SUPPORTING INFORMATION

Supporting information for this article is available at https://doi.org/10.1162/netn_a_00128.

## AUTHOR CONTRIBUTIONS

Shota Shirai: Data curation; Formal analysis; Investigation; Software; Writing - Original Draft. Susant Kumar Acharya: Data curation; Formal analysis; Investigation; Methodology; Writing - Original Draft; Writing - Review & Editing. Saurabh Kumar Bose: Data curation; Formal analysis; Methodology. Joshua Brian Mallinson: Data curation; Formal analysis; Investigation; Methodology; Writing - Review & Editing. Edoardo Galli: Data curation; Formal analysis; Methodology. Matthew D. Pike: Investigation; Methodology; Software. Matthew D. Arnold: Formal analysis; Methodology; Software. Simon Brown: Conceptualization; Formal analysis; Funding acquisition; Investigation; Methodology; Project administration; Resources; Supervision; Validation; Writing - Original Draft; Writing - Review & Editing.

## FUNDING INFORMATION

Simon Brown, MacDiarmid Institute for Advanced Materials and Nanotechnology (http://dx.doi.org/10.13039/501100013266). Saurabh Kumar Bose, Marsden Fund (http://dx.doi.org/10.13039/501100009193). Simon Brown, Ministry of Business, Innovation, and Employment (http://dx.doi.org/10.13039/501100003524), Award ID: UOCX1603.

## TECHNICAL TERMS

Neuromorphic computing: Computation that is inspired by biological principles, especially those of the mammalian brain.
Scale-free topology: A network topology in which the number of connections per node is power-law distributed.
Reservoir computing: A computational framework that utilizes a nonlinear dynamical system (a reservoir) to project inputs into a higher dimensional space, allowing mapping to desired outputs by training a single linear readout layer.
Self-similar temporal dynamics: A temporal structure that has similar characteristics on multiple timescales.
Long-range temporal correlations: Correlations characterized by slowly power-law-decaying autocorrelation function in temporal domain.
Criticality: A dynamical state that is poised at a phase transition, and that exhibits long-range spatial and temporal correlations.
Tunnel gaps: A sub-nanometer gap between two conducting bodies in which electronic transport can occur via quantum tunneling.
Interevent interval (IEI): Time between two consecutive events.
