# Supplementary material for: Long-range temporal correlations in scale-free neuromorphic networks
Source: Netw Neurosci. 2020 Apr 1;4(2):432–47. doi: 10.1162/netn_a_00128 (PMC7286302; doi:10.1162/netn_a_00128)
Supplement: Supplementary file 1 [file netn-04-432-s001.pdf]

RESEARCH

Shirai, S., Acharya, S. K., Bose, S. K., Mallinson, J. B., Galli, E., Pike, M. D., Arnold, M. D., & Brown, S. A. (2020). Supporting information for "Longrange temporal correlations in scale-free neuromorphic networks." *Network Neuroscience*, *4*(2), 432–447. https://doi.org/10.1162/netn_a_00128

Supplementary Materials: Long-range temporal correlations in scale-free neuromorphic networks

Shota Shirai[1,a], Susant Kumar Acharya[1,a], Saurabh Kumar Bose[1], Joshua Brian Mallinson[1], Edoardo Galli[1], Matthew D. Pike[2], Matthew D. Arnold[3] and Simon Anthony Brown[1,*]

[1]The MacDiarmid Institute for Advanced Materials and Nanotechnology, School of Physical and Chemical Sciences, Te Kura Matū, University of Canterbury, Private Bag 4800, Christchurch 8140, New Zealand

[2]Electrical and Electronics Engineering, University of Canterbury, Private Bag 4800, Christchurch 8140, New Zealand

[3]School of Mathematical and Physical Sciences, University of Technology Sydney, Australia

[*]Corresponding author (simon.brown@canterbury.ac.nz)

[a]S.S and S.K.A contributed equally to this work

NUMERICAL SIMULATION OF PERCOLATING TUNNELLING NETWORK

Numerical simulations of two dimensional percolating tunnelling networks can give valuable insight into the structure in networks of nanoparticles, which are well described by continuum percolating tunnelling models (Fostner, Brown, Carr, & Brown, 2014). In continuum percolation, the conducting objects are uniform discs (representing the deposited particles in our experiments). The discs, shown schematically in Figures 1 and 2, land randomly and are allowed to overlap with each other when they land (representing coalescence between neighbouring particles and formation of groups) (Fostner et al., 2014; Fostner & Brown, 2015). In this regime, with surface coverage $p < p_c$, i.e. below the percolation threshold, no single group spans the entire network and the conduction of the system is due to tunnel currents flowing across small gaps which separate the groups of particles. Each gap is assigned a conductance, $G_i = A\exp(-\delta L_i)$, where A and δ are constants and L_i is the size of the gap ($A = 1$ and $\delta = 100$ for convenience) (Fostner et al., 2014). Here it is assumed that the groups are large enough that both the charging energy of a connected group and the quantization of energy levels are negligible, and that the resistance between overlapping particles within a group is negligible, so that the only resistance in the system is due to tunnelling between groups. We focus primarily on simulations with a system size of 200×200 particle diameters (chosen to provide the best trade-off between computational time and

finite-size effects) and surface coverage $p < p_c$. However these parameters are varied independently to investigate finite size effects (see below) and the effect of particle density (Figure 3 A-C). We also counted the number of connections from each node (group of particles) to other nodes (Figure 3 D-F), which is called the degree of the node, k (Barabási & Oltvai, 2004). We also computed the distributions of the group sizes, g, and of k (Figure 4), as well as clustering coefficients, and the average length of the shortest paths between all pairs of nodes (the average shortest path length), as described below.

Finite Size Effect on Numerical Simulation

As explained in the main text, our simulation results show that the degree distribution of the network at the percolation threshold is heavy-tailed and is close to a power-law with exponent 2.5, indicating the existence of a scale-free network. However, the degree distribution is constrained by the finite size of the network, which imposes a cut-off on the tail of the power-law distribution (Waclaw & Sokolov, 2007). To account for finite size effects, we simulated various system sizes in our numerical simulations (Figure 5A) at a surface coverage of 0.65. At lower system sizes of 50×50 and 100×100 particle diameters, there is a cut-off in the tail of the power-law distribution. But, for system sizes of 200×200 particle diameters and above, finite-size effects are negligible and do not have significant impact on the exponent φ. Note that the experimental system is much larger with a spacing between contacts of $\sim 5 \times 10^3$ particle diameter.

Statistical Properties of the Network

For complex networks, the average shortest path length and clustering coefficient can give significant insight into the underlying network architecture (Albert & Barabási, 2002; Ravasz & Barabási, 2003; Strogatz, 2001). The average shortest path length $\langle L \rangle$ is the average number of connections separating any two nodes in the network. We obtained $\langle L \rangle \sim 6$ for a surface coverage of 0.65, and it decreases monotonically with increase in coverage upto 0.68 (Figure 5B). With increasing coverage, the number of high degree nodes increases monotonically (see Figure 1E in the main text) providing connectivity between other low degree nodes at remote locations in the network. Conversely, $\langle L \rangle$ increases monotonically with increasing system size as shown in Figure 5C. This is because with fixed coverage, increasing the system size increases the total number of nodes in the network, but does not increase the relative number of high degree nodes. Therefore, the average path length between any two nodes will be

larger for larger system sizes of the same coverage. Nonetheless, $\langle L \rangle$ is very small compared with the total number of nodes, which is a typical characteristic of a small-world networks (Strogatz, 2001).

The clustering coefficient quantifies the number of connections that exist between the nearest neighbors of a node as a proportion of the maximum possible number of connections between the neighbors. Mathematically, for node i with k_i links, the clustering coefficient $C_i = 2E_i/k_i(k_i - 1)$, where E_i is the number of links among the neighbors of i (Albert & Barabási, 2002; Ravasz & Barabási, 2003). The average clustering coefficient is simply given by $\langle C \rangle = \sum_i C_i/N$, where N is the total number of nodes. For our network, $\langle C \rangle$ is around 0.52 for surface coverage of 0.65 (Figure 5B), pointing to the existence of a highly modular structure (Ravasz & Barabási, 2003). $\langle C \rangle$ varies in a narrow range (between 0.49 to 0.53) as a function of system size as shown in (Figure 5C), showing that the finite-size effects have negligible influence on the clustering coefficient.

To establish the existence of small-world properties in our networks, we compared $\langle L \rangle$ and $\langle C \rangle$ to the estimates for random and regular networks of equivalent size and degree distribution as outlined in ref. (Watts & Strogatz, 1998). Generally, $\langle L \rangle$ ranges from approximately $\ln(N)/\ln(k)$ for random networks to $\sim N/2k$ for regular networks, whereas $\langle C \rangle$ ranges from approximately $\sim k/N$ to 0.75. Small-world networks are typically characterized by significantly higher $\langle C \rangle$ than random networks, yet they have $\langle L \rangle$ comparable to random networks (Albert & Barabási, 2002; Barabási & Oltvai, 2004; Watts & Strogatz, 1998). For the networks shown in Figure 5B (size 200×200, coverage 0.64 to 0.68), the average degree $\langle k \rangle \sim$ 3.5 and $1 \times 10^3 < N < 2 \times 10^3$. Hence $\langle L \rangle \sim$ 6 which is close to the value expected for the random network (*cf.* 3×10^2 for the regular one), and $\langle C \rangle \sim$ 0.6 which is close to the value expected for the regular network (*cf.* 2×10^{-2} for the random one). Taken together with the scale-free degree distribution shown in Figure 1E in the main text and Figure 5A, these data strongly suggest that our nanoparticle networks have small-world attributes.

A hierarchical network is a network which has a scale-free topology and a high degree of clustering (Barabási & Oltvai, 2004; Ravasz & Barabási, 2003). The quantifiable signature of a hierarchical network is the dependence of clustering on degree, $C(k)$, which follows $C(k) \sim k^{-1}$ (Ravasz & Barabási, 2003). To find out if hierarchical organization is present in our nanoparticle network, we calculated $C(k)$ for various system sizes. As shown in Figure 5D, $C(k)$ scales as k^{-1} for all system sizes, indicating that our nanoparticle networks have hierarchical architecture. This indicates that the higher a node's connectivity, the smaller its degree of clustering and vice versa.

Event Detection and Effect of Threshold Variation on IEI and ACF

The upper panel of Figure 7A shows a typical time trace of conductance (G) with the response to constant applied voltage, showing switching between discrete conductance states. The middle panel shows the corresponding changes in conductance ΔG. A threshold (black lines in the ΔG trace) is used to identify the switching events, i.e. ΔG greater than the threshold are counted as events. The value of the threshold is chosen to be close to but above the noise level. The identified switching events are represented as an event-train, as shown in the bottom panel. A consequence of using a threshold is that some small events of comparable size to the noise band will not be detected. Here we show that for threshold values in a reasonable range, the effect of missed events is negligible.

In order to investigate the effect of the thresholds in the IEI distributions and ACFs, the thresholds were varied from $2\mathrm{mG}_0$ to $5\mathrm{mG}_0$ and the fitting procedure described earlier was applied to the IEI distributions for each threshold. Figure 7B shows that the IEI distributions are very robust against variation of the threshold and pass the KS test for more than 3 orders of magnitude of IEI. Figure 7C shows that the IEI exponents did not show significant changes as a function of threshold.

As shown in Figure 7D, ACFs for all examined thresholds show power-law behavior (red lines) with slope smaller than the slopes measured for uncorrelated data (grey lines), indicating that the LRTC behavior is robust against variation of threshold. The correlation strength (the ACF at lag-2) and ACF slopes varies within a narrow range (see Figure 7E).

The robustness of the analysis against the threshold selection can be interpreted as a consequence of the self-similar switching activity as shown in Figure 6 and Figure 3A in the main text.

MODELLING OF CORRELATION

Hierarchical Burst Model

The switching activity pattern in our neuromorphic network is bursty on multiple time scales as shown in Figure 2A in the main text. We define a burst as a collection of successive switching events with IEIs $<$ $\mathrm{IEI}_{\mathrm{mean}}$ (the mean IEI value) (Friedman et al., 2012). Each successive burst is separated by IEIs $>$ $\mathrm{IEI}_{\mathrm{mean}}$ (see Figure 9A for the defined bursts in shades of orange). The bursts are characterized by the distribution of the burst size (S) (Jo, 2017; Karsai, Kaski, Barabási, & Kertész, 2012) which is the number of events in a single burst. The burst size distribution in our experimental data typically follows a power-law with

the exponent of $\sim$2, as shown in Figure 9B. As mentioned in the main text, LRTC in our devices is believed to originate from a hierarchy of bursts (Linkenkaer-hansen, Nikouline, Palva, & Ilmoniemi, 2001). We now model this hierarchical burst time series as described below.

The simulated time series is constructed from the power-law distributed IEIs and burst sizes, both are generated using the power-law distributed random number generator (Clauset, Shalizi, & Newman, 2009; Marshall et al., 2016). The power-law exponents are set to be 1.8 for IEIs and 2 for the burst sizes, based on the typical experimental result shown in Figure 3B in the main text and Figure 9B. In the hierarchical IEI model, generated burst sizes serve as the top most layer of the hierarchy, shown as S_1 in Figure 9C. Accordingly, these bursts are separated by the longest intervals from the IEI distribution, which are shaded in red in the Figure 9C. The second layer S_2 of the hierarchy is created by subdividing every burst in S_1 (with $S > 1$) into smaller bursts by drawing again from the same power law used to generate S_1. The bursts in S_2 are separated by IEIs which are shorter than those used to separate the bursts in S_1, which are shaded in blue in Figure 9C. From one layer to the next, bursts are partitioned into smaller and smaller bursts which are separated by smaller and smaller intervals. This process is repeated until the n_{th} layer features no bursts with $S > 1$. It is important to note that this model does not change the number of events or the IEI distribution, it only arranges the order of successive IEIs. The sampling interval in the simulation is set to be the same sampling rate as in Figure 3A in the main text.

Figure 9D shows event-trains that were generated using the hierarchical burst model on multiple time scales. The temporal patterns are similar at different scales and are qualitatively similar to those of the experimental data as shown in Figure 6 and Figure 3A in the main text. As shown in Figure 3C in the main text, the ACF for this hierarchical burst model qualitatively matches the experimental ACF, while the bursty time series without hierarchical arrangement of the IEIs (green line in Figure 9E) does not: it decays faster than the ACF of the hierarchical burst model. The essential point of this modelling is that hierarchical bursting is required in order to reproduce the correlations in the experiments.

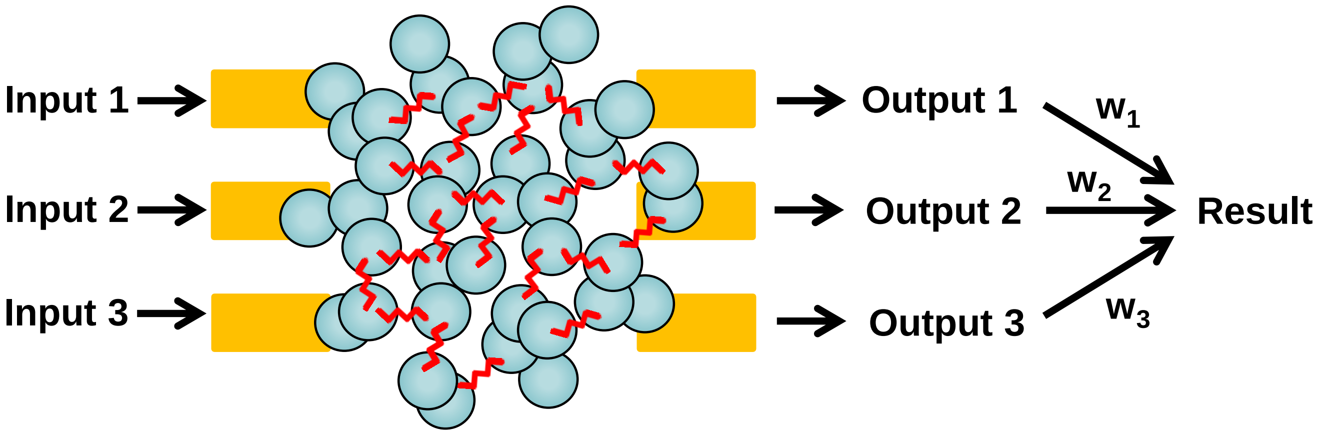

Figure 1. Illustration of the reservoir computing concept (Lukoševičius & Jaeger, 2009; Maass et al., 2002) using a percolating tunnelling network of nanoparticles. Input signals are fed into the reservoir (i.e. network) via a number of electrodes. A combination of non-linearity, memory and recurrency in the network transforms the signals (“into a higher dimensional space” in the jargon of this field). The various transformed signals are read out through a set of output electrodes. The output signals, x_i, will be more complex functions that are influenced by previous states inside the reservoir. A training procedure is then used to choose weights that (in a standard classification task) achieve as closely as is possible a sum $y = \sum w_i x_i$ that is equal to a different target value for each type of input. For example a standard classification task is to distinguish sine and square wave inputs (Riou et al., 2019; Torrejon et al., 2017), in which case the weights are chosen to achieve values $y = 0$ and $y = -1$ for the sine and square inputs respectively. After training, new test data is fed into the reservoir and the same weight values are used to classify the new data. The success of the classification procedure is then evaluated.

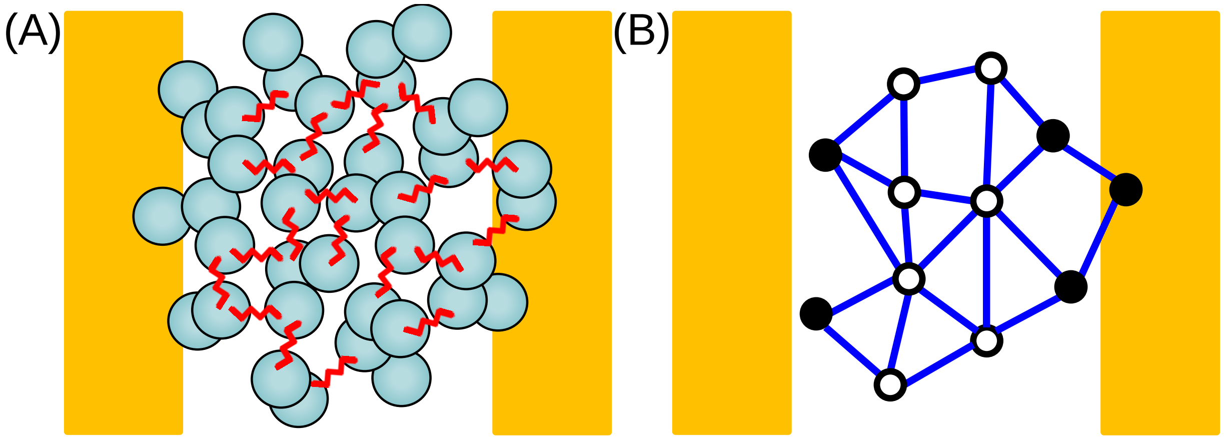

Figure 2. (A) Schematic illustration of the percolating tunnelling network and the formation of groups of nanoparticles during deposition. Tunnel gaps, which are the active sites for atomic scale switching processes, are illustrated with red resistor symbols. (B) The equivalent network in the form shown in Figure 1 (D) in the main text. The circles represent the centres of mass of the groups and blue lines indicate the connections between groups (i.e. the tunnel gaps that connect groups). Filled circles represent groups of particles that are connected to the electrodes.

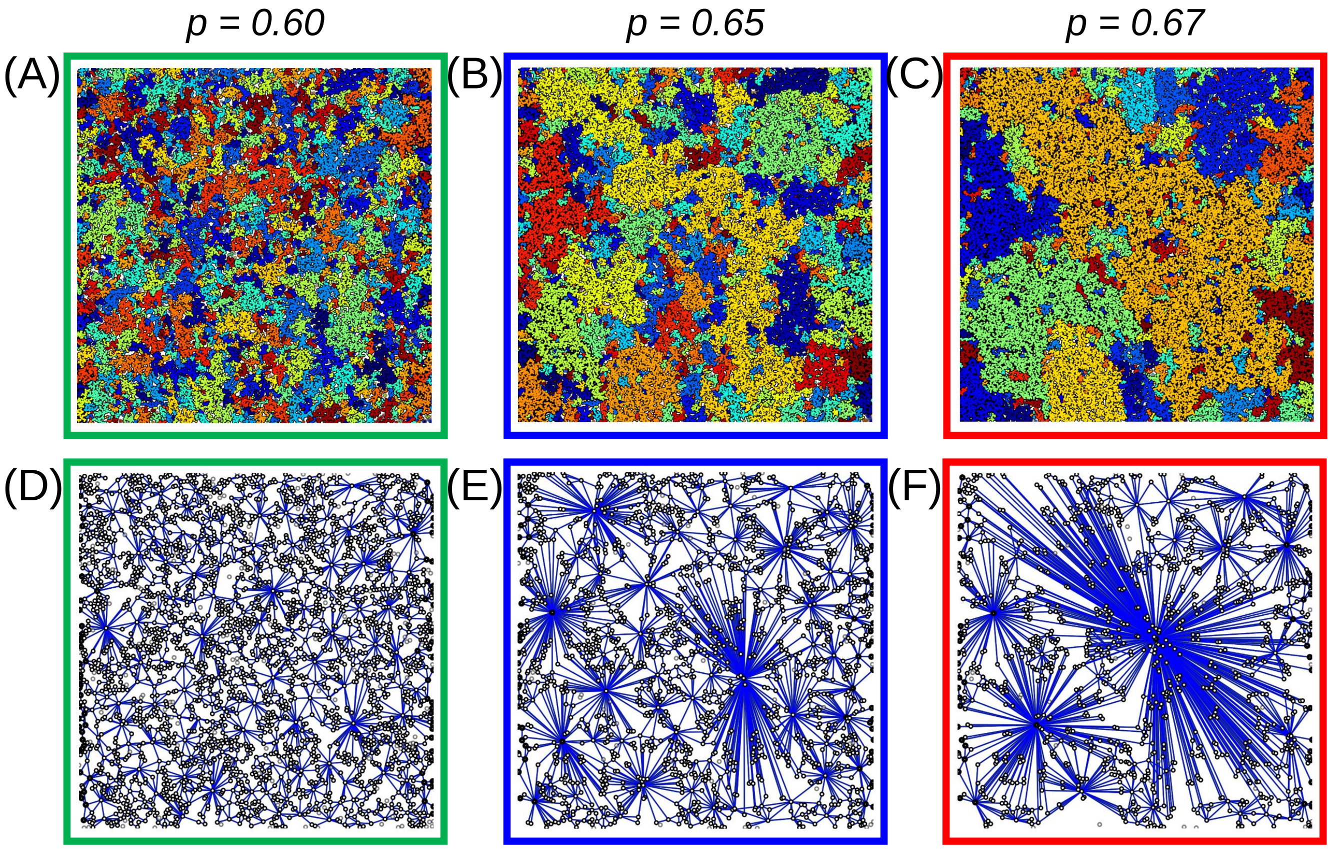

Figure 3. Numerical simulation for a system size of 200 $\times$ 200 particle diameters for different surface coverages: (A) $p = 0.60$ ($p \ll p_c$), (B) $p = 0.65$ ($p < p_c$), and (C) $p = 0.67$ ($p \sim p_c$). (D) to (F) show the corresponding maps of the connections between groups corresponding to (A) to (C). For $p \ll p_c$, there are only small groups of particles, for $p < p_c$ bigger groups of particles appear, and for $p \sim p_c$ a few large groups are formed.

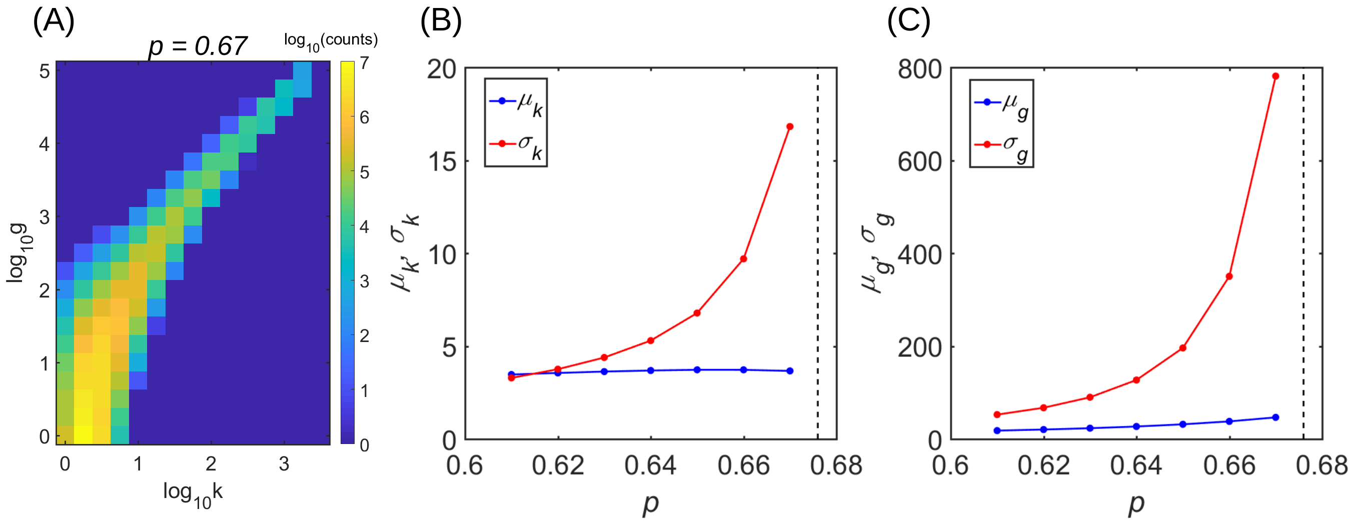

Figure 4. (A) Distribution of the sizes of groups (specifically g, the number of particles in each group) and degree of each group (specifically k, number of connections to neighbouring groups), for the case of surface coverage $p = 0.67$. Distributions for lower values of p are similar but have a narrower range of both g and k. (B) Dependence of the mean value of k (μ_k) and the standard deviation of k (σ_k) on surface coverage p. (C) Corresponding plot of the mean value of g (μ_g) and the standard deviation of g (σ_g) on surface coverage p. The key feature of the plots is the broadening of the distributions (increase in σ) as p approaches p_c, consistent with divergence of the correlation length at p_c (Schmelzer et al., 2002; Stauffer & Aharony, 2003).

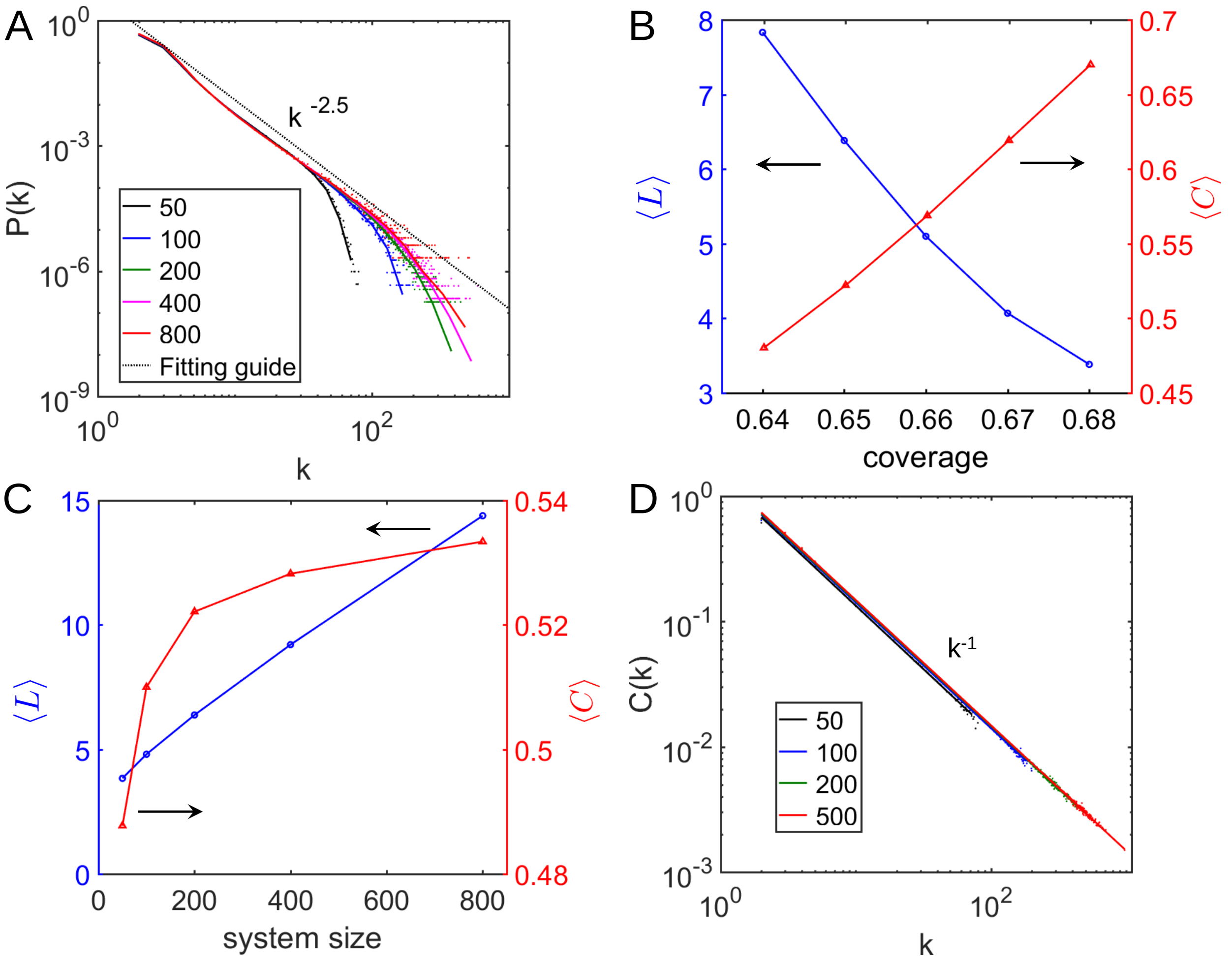

Figure 5. Finite-size effect on numerical simulations and statistical properties of percolating tunneling network. (A) The PDF of degree distribution (k) at a surface coverage of 0.65 for various system sizes (50×50, 100×100, 200×200, 400×400 and 800×800) with each color corresponding to a different system size. The tail of the degree distribution ($P(k)$) is not significantly affected beyond the system size of 200×200 particle diameters. The black dotted line is a guide to the eye corresponding to an exponent of 2.5. (B) The evolution of average path length (blue line) and clustering coefficient (red line) with surface coverage at a system size of 200×200 particle diameters. The average path length decreases, while the clustering coefficient increases monotonically with increase in surface coverage. (C) The variation of the average path length (blue line) and clustering coefficient (red line) with system size at a surface coverage of 0.65. The average path length continuously increases with increase in system size, while the clustering coefficient increases only slightly upto a system size of 200×200 particle diameters and then saturates. High clustering coefficient and low average path length in our nanoparticle network is indicative of a small-world architecture. (D) The variation of average clustering coefficient per degree for various system sizes (50×50, 100×100, 200×200 and 500×500). The clustering coefficient per degree distribution decays with a power-law exponent of 1, suggesting the existence of a hierarchical architecture in the percolating tunneling network (Albert & Barabási, 2002; Ravasz & Barabási, 2003).

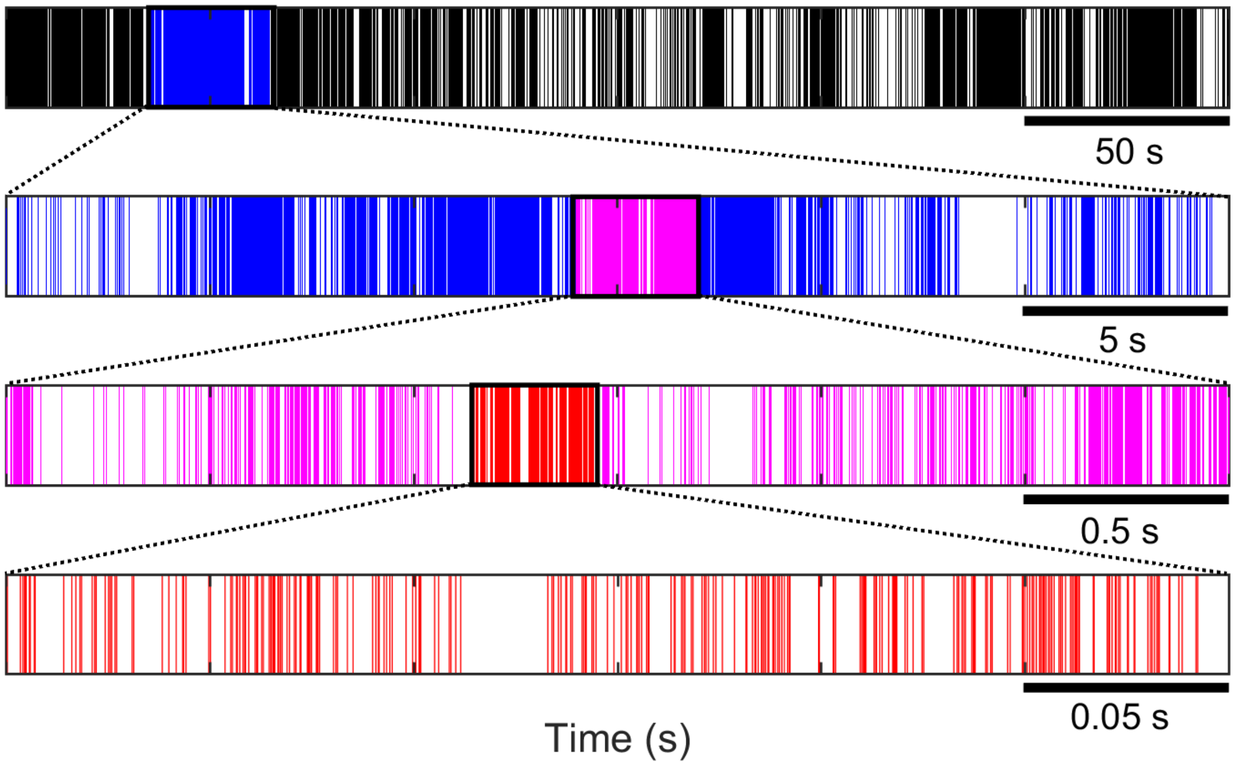

Figure 6. Self-similar switching patterns for fast measurement (200 μs sampling rate), showing that the self-similarity is almost identical to that shown for slow measurements in Figure 3A in the main text. The top panel shows a section of signal comprising 500 s of data, while the subsequent panels show sections of the top panel with the temporal scale magnified 10, 100 and 1000 times. The switching activity patterns in the four panels is not readily distinguishable, which indicates that the switching events on different time scales qualitatively are self-similar.

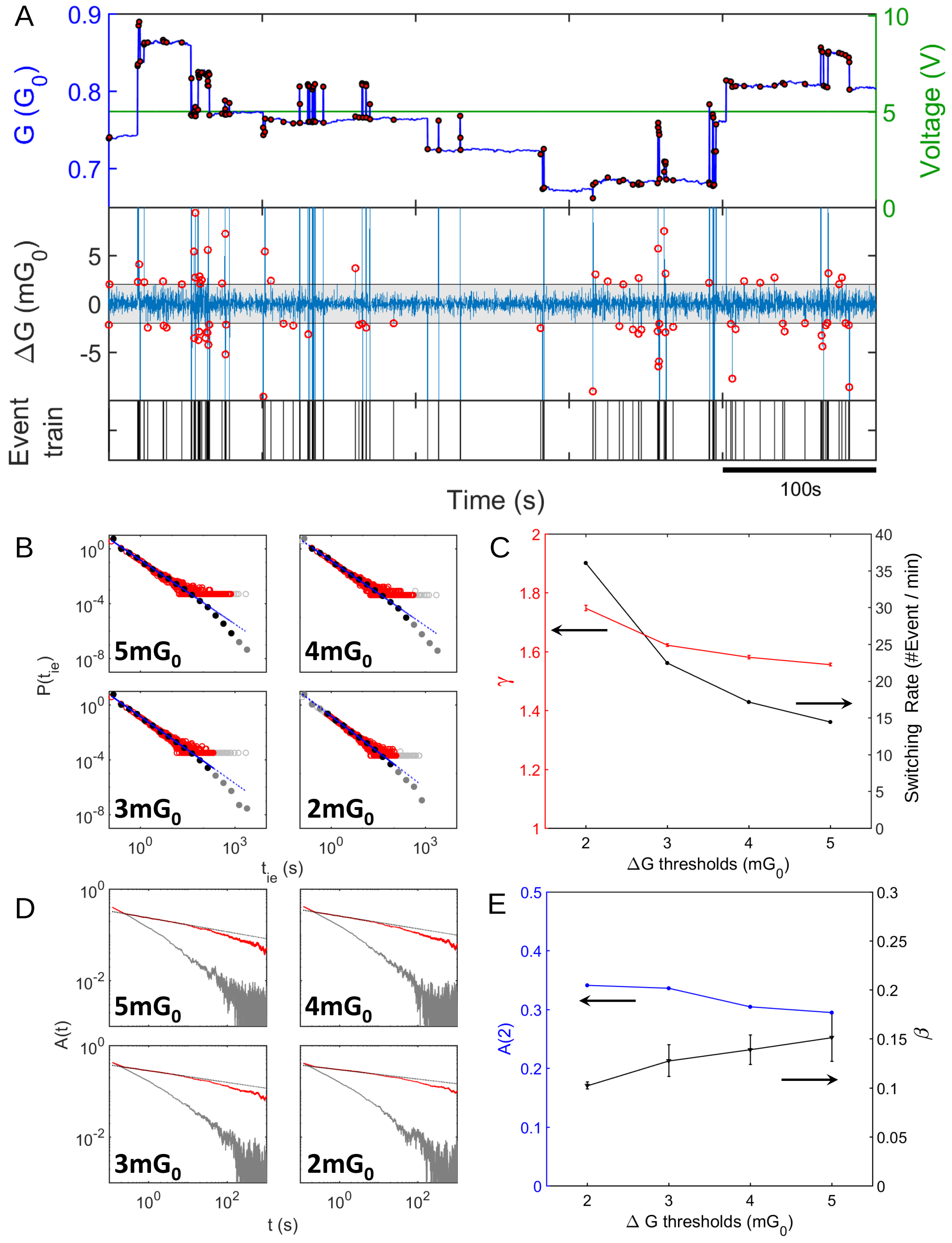

Figure 7. Event detection procedure and the effect of threshold variation on IEI distribution and ACF. (A) The temporal evolution of the device conductance (G: top panel) and the change in G (ΔG: middle panel) with detected events (red markers). Signals $|\Delta G|$ above the selected threshold (black lines in the middle panel) are detected as switching events and recorded in the event-train (bottom panel). The grey shaded region in the middle panel represents the noise band. (B) The IEI distribution obtained for a range of thresholds between 2mG_0 and 5mG_0. All the IEI distributions pass the KS test over more than 2 orders of magnitude in time. (C) The estimated power-law exponents are within the expected range of 1.7 ± 0.1, even though the switching rate changes significantly. (D) The ACF exhibits a slow power-law decay for a wide range of threshold selections. (E) The correlation strength at lag-2 (A(2)) (blue line) and the ACF slope (black line) do not change within the fitting uncertainties.

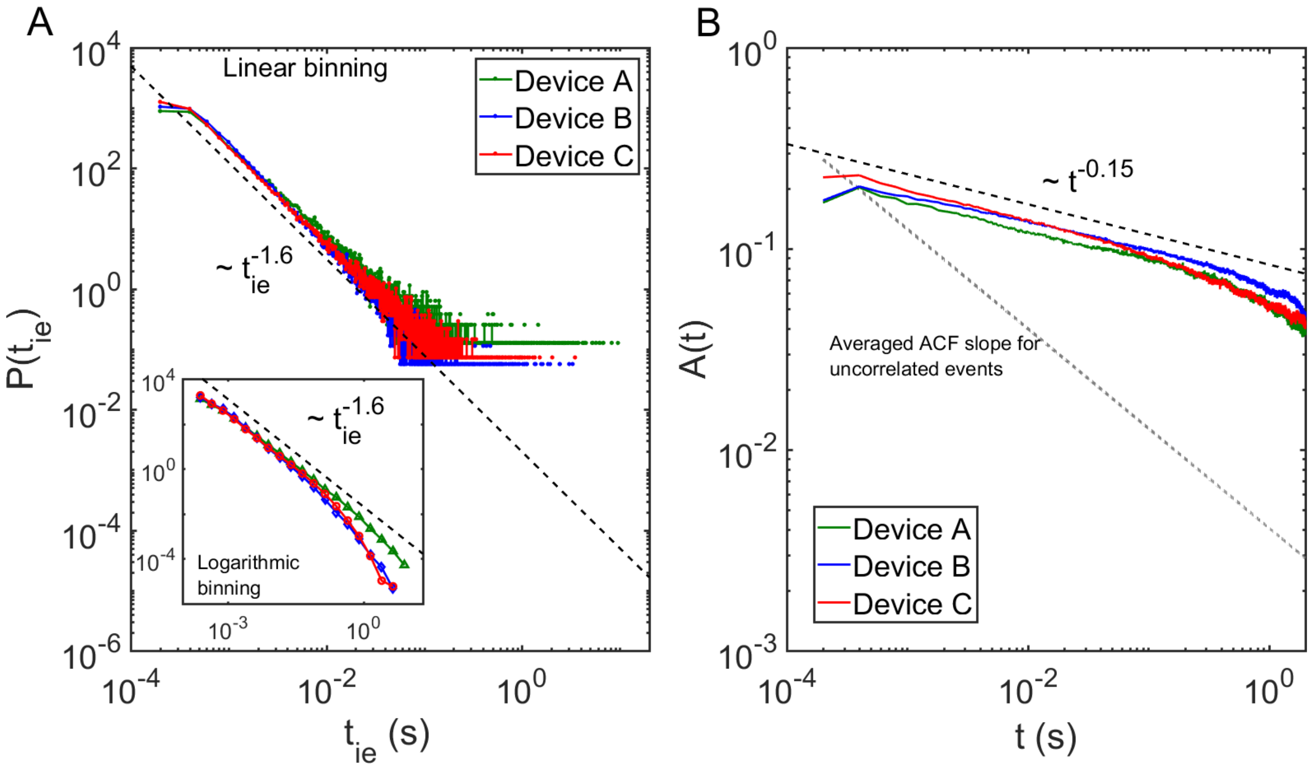

Figure 8. Device dependence of the IEI distribution and the ACF. (A) The IEI distributions for three different devices at the same voltages. While the cut-off of the tail are slightly different (inset figure; logarithmic binning), the slopes of the distributions are almost identical (see with the guide line of $\gamma = 1.6$). (B) The ACFs also show LRTC with similar decay exponents. The experimental decay slopes (dashed line) are much lower than the ACF slope of the uncorrelated events (dotted line).

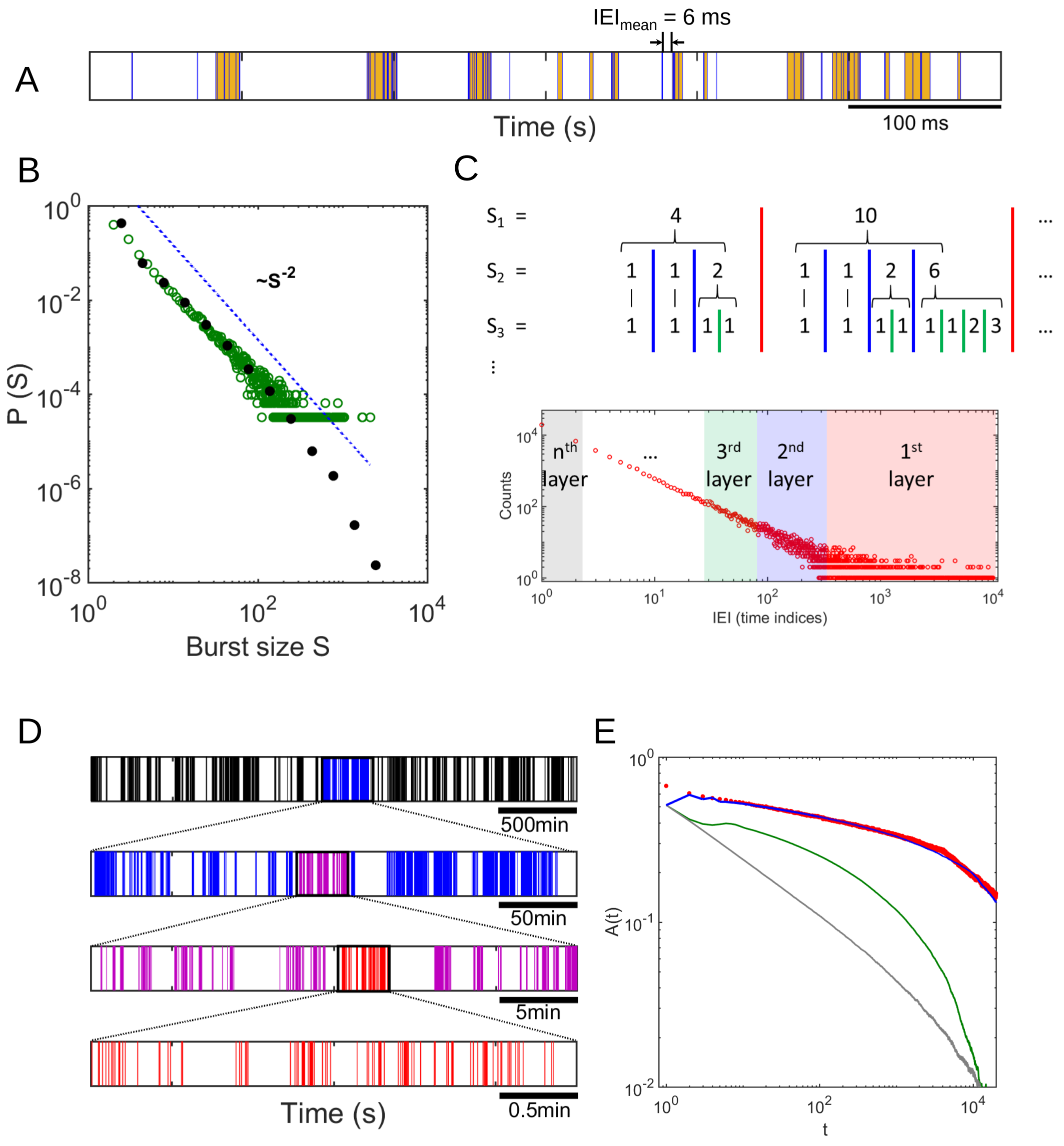

Figure 9. The bursty activity pattern in our experimental data and the hierarchical burst model. (A) The bursts (highlighted in orange) are collections of successive events separated by IEIs less than the mean IEI (IEI_{mean} = 6 ms). (B) The burst size distribution ($P(S)$) follows a power-law for more than 3 orders of magnitude of size with the exponent $\sim$ 2. (C) Schematic of the hierarchical burst model. The burst in the layer n has a further burst structure created in layer $n+1$ i.e. the bursts are themselves made up of bursts. The created burst is separated by smaller IEIs in each successive layer. (D) The event-trains for different time scales in the hierarchical burst model. The event-trains feature bursty patterns which are qualitatively the same across different time scales, demonstrating that large bursts of activity are comprised of smaller bursts of activity, i.e. the bursts of activity are temporally self-similar. This self-similar structure induces the LRTC in the ACF. (E) Only the ACF of the hierarchical burst model (blue line) can successfully replicate the experimental ACF (red markers). Although the non-hierarchical burst model (green line) has a smaller slope than the ACF of the uncorrelated IEI (gray line), the slope is much greater than that obtained from the hierarchical burst model.

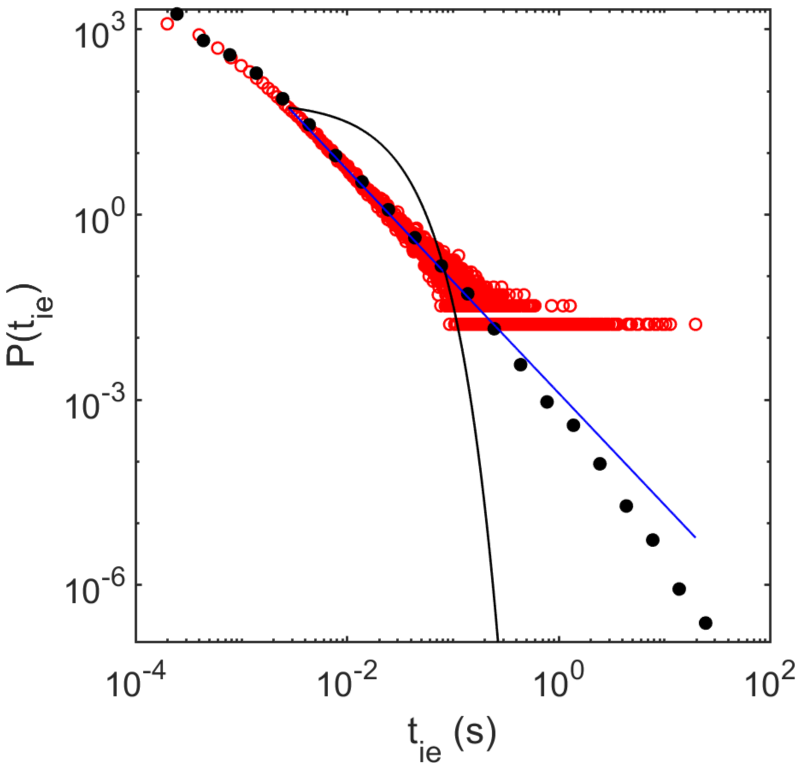

Figure 10. The result of MLE for power-law (blue line) and exponential (black line) fits to the IEI distribution in Figure 3B in the main text. The fitting range for the exponential is set to be the same range of the power-law. While the power-law passes the KS test (p-value > 0.2) with more than 2 orders of magnitude in time, the exponential distribution fails the KS test (p-value < 0.01).

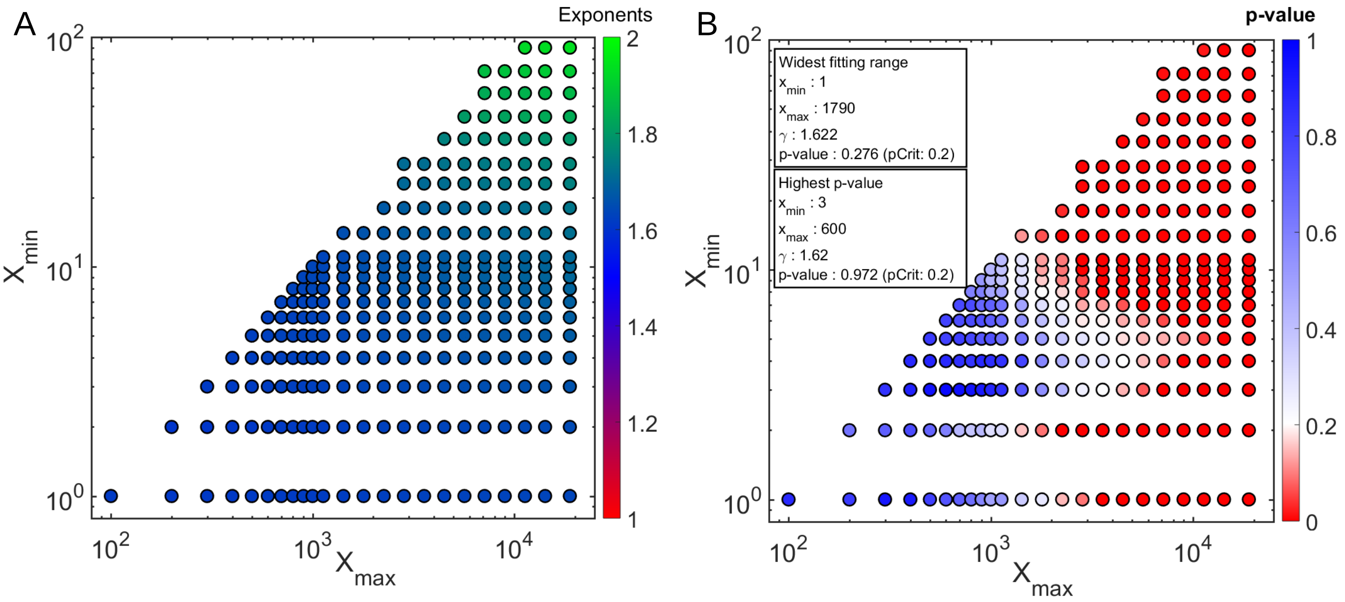

Figure 11. Maps of exponents estimated by MLE and corresponding p-values from the KS test when the distributions are truncated with various lower ($\mathrm{X_{min}}$) and higher ($\mathrm{X_{max}}$) cut-off values, following the procedures developed in ref. (Marshall et al., 2016). See supplementary text for further discussion. (A) Map of exponents obtained by MLE for different ($\mathrm{X_{min}}$) and ($\mathrm{X_{max}}$). The exponents range between 1.5 and 2 for all selected ($\mathrm{X_{min}}$) and ($\mathrm{X_{max}}$). The exponents do not significantly change for different cut-offs. (B) Associated p-values obtained from KS test indicate the probability that the null hypothesis for power-law can be rejected. Blue and white dots indicate plausibility of the power-law hypothesis (p-value > 0.2), while red dots suggest the power-law hypothesis is not plausible (p-value < 0.2). A wide range of the distributions (> 3 orders of magnitude in time) are found to be consistent with the power law hypothesis (p-value > 0.2). The best fit exponent is chosen in accordance with the widest range of the distribution that passes the KS test.

REFERENCES

Albert, R., & Barabási, A.-L. (2002). Statistical mechanics of complex networks. *Reviews of Modern Physics*, *74*(1), 47–97.

Barabási, A.-L., & Oltvai, Z. N. (2004). Network biology: understanding the cell's functional organization. *Nature Reviews Genetics*, *5*(2), 101–113.

Clauset, A., Shalizi, C. R., & Newman, M. E. J. (2009). Power-law distributions in empirical data. *SIAM Review*, *51*(4), 661–703.

Fostner, S., Brown, R., Carr, J., & Brown, S. A. (2014). Continuum percolation with tunneling. *Physical Review B*, *89*(7), 075402.

Fostner, S., & Brown, S. A. (2015). Neuromorphic behavior in percolating nanoparticle films. *Physical Review E*, *92*(5), 052134.

Friedman, N., Ito, S., Brinkman, B. A., Shimono, M., Deville, R. E., Dahmen, K. A., … Butler, T. C. (2012). Universal critical dynamics in high resolution neuronal avalanche data. *Physical Review Letters*, *108*(20), 208102.

Jo, H.-H. (2017). Modeling correlated bursts by the bursty-get-burstier mechanism. *Physical Review E*, *96*(6), 062131.

Karsai, M., Kaski, K., Barabási, A.-L., & Kertész, J. (2012). Universal features of correlated bursty behaviour. *Scientific Reports*, *2*, 397.

Linkenkaer-hansen, K., Nikouline, V. V., Palva, J. M., & Ilmoniemi, R. J. (2001). Long-range temporal correlations and scaling behavior in human brain oscillations. *The Journal of Neuroscience*, *21*(4), 1370–1377.

Lukoševičius, M., & Jaeger, H. (2009). Reservoir computing approaches to recurrent neural network training. *Computer Science Review*, *3*(3), 127–149.

Maass, W., Natschläger, T., & Markram, H. (2002). Real-time computing without stable states: A new framework for neural computation based on perturbations. *Neural Computation*, *14*(11), 2531–2560.

Marshall, N., Timme, N. M., Bennett, N., Ripp, M., Lautzenhiser, E., & Beggs, J. M. (2016). Analysis of power laws, shape collapses, and neural complexity: New techniques and MATLAB support via the NCC toolbox. *Frontiers in Physiology*,

7, 250.

Ravasz, E., & Barabási, A.-L. (2003). Hierarchical organization in complex networks. *Physical Review E*, *67*(2), 026112.

Riou, M., Torrejon, J., Garitaine, B., Abreu Araujo, F., Bortolotti, P., Cros, V., … Grollier, J. (2019). Temporal Pattern Recognition with Delayed-Feedback Spin-Torque Nano-Oscillators. *Physical Review Applied*, *12*(2), 024049.

Schmelzer, J., Brown, S. A., Wurl, A., Hyslop, M., & Blaikie, R. J. (2002). Finite-size effects in the conductivity of cluster assembled nanostructures. *Physical Review Letters*, *88*(22), 226802.

Stauffer, D., & Aharony, A. (2003). *Introduction to Percolation Theory*. Taylor & Francis.

Strogatz, S. H. (2001). Exploring complex networks. *Nature*, *410*, 268–276.

Torrejon, J., Riou, M., Araujo, F. A., Tsunegi, S., Khalsa, G., Querlioz, D., … Grollier, J. (2017). Neuromorphic computing with nanoscale spintronic oscillators. *Nature*, *547*(7664), 428–431.

Waclaw, B., & Sokolov, I. M. (2007). Finite-size effects in Barabási-Albert growing networks. *Physical Review E*, *75*(5), 056114.

Watts, D., & Strogatz, S. (1998). Collective dynamics of small-world' networks. *Nature*, *393*(6684), 440–442.
